# Meiofauna Biodiversity and Community Structures in the Barents Sea and Nansen Basin Are Influenced by Benthic Spatiotemporal Heterogeneity

**DOI:** 10.1002/ece3.72777

**Published:** 2026-03-11

**Authors:** Joel Vikberg Wernström, Bodil A. Bluhm, Katarzyna Grzelak, Anders Hobæk, Doris Björling, Andreas Altenburger

**Affiliations:** ^1^ The Arctic University Museum of Norway, UiT The Arctic University of Norway Tromsø Norway; ^2^ Department of Arctic and Marine Biology UiT The Arctic University of Norway Tromsø Norway; ^3^ Institute of Oceanology, Polish Academy of Sciences Sopot Poland; ^4^ Norwegian Institute for Water Research (NIVA) Bergen Norway; ^5^ Department of Marine Sciences Gothenburg University Göteborg Sweden

**Keywords:** Arctic, biodiversity, community ecology, meiobenthos, seasonality

## Abstract

Meiobenthic metazoans occur worldwide and are a cornerstone of aquatic ecosystems. Here, we investigate the composition of meiobenthic communities along a transect in the Barents Sea and Nansen Basin and provide the first meiobenthos dataset for this region to combine seasonal resolution with high taxonomic fidelity. We identified 12 higher meiobenthic taxa and provide identifications of 143 nematode genera, 37 copepod genera, 11 species of kinorhynchs, the meiobenthic hydrozoan *Plotocnide*, and the first loriciferan records for the region. Nematodes dominated communities (47%–90% of abundances), followed by copepods (3%–24%) and kinorhynchs (≤ 10%), and we provide a comprehensive taxonomic dataset of these groups along with analyses of structuring environmental and spatiotemporal factors. We observed a general decrease in abundances and biodiversity with increasing water depth, and high richness of several taxa around the northern shelf and continental slope. Community structuring appears to be driven primarily by spatial effects on small and large scales in addition to variation across the four seasons, while granulometric properties and organic carbon levels of the sediment also have a demonstrable effect on nematode, copepod and kinorhynch communities. Based on our analyses, we draw the conclusion that these spatiotemporal and environmental factors coalesce to influence community structures of meiobenthic metazoans in the Barents Sea. While our synthesis of environmental and biodiversity data represents a contribution to the knowledge of current Arctic metazoan meiobenthos communities, they are vulnerable to alterations resulting from drastic climate‐driven changes in the near future and require further study.

## Graphical abstract

Benthic meiofauna in the Barents Sea were investigated for spatiotemporal variability and taxonomic composition.

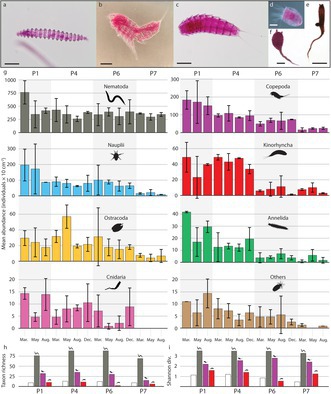

## 1 Introduction

Tiny bottom‐dwelling invertebrates, also known as metazoan meiobenthos or meiofauna, form an important component of marine ecosystems because of their ubiquity and diversity. Over the past five decades, research has emphasised the ecological roles played by the meiobenthos (Schratzberger and Ingels 2018), which include production, consumption and decomposition of organic matter, regeneration of nutrients, energy transfer to higher trophic levels, and bioturbation on a massive scale (Coull 1990; Rysgaard et al. 2000; Majdi et al. 2020; Ptatscheck et al. 2020). Forecasted environmental changes could have outsized impacts on meiobenthic communities, and especially so in the Arctic (Zeppilli et al. 2015; Leasi et al. 2021), precipitating a need for more detailed biodiversity and ecological data from this region. Abundant clades of marine meiobenthos include taxa such as nematodes, harpacticoid copepods and kinorhynchs, and these groups usually dominate sublittoral communities (Vanreusel et al. 2000; Lee et al. 2003; Leasi et al. 2021). Nematodes are the most abundant meiobenthic clade overall (Pfannkuche and Thiel 1987). This is no less true for the Arctic seas; in the Fram Strait, for example, they represent up to 97% of the total meiobenthos abundance (Schnier et al. 2023). On the Barents Sea shelf, nematodes can comprise a slightly lower, but still dominant 89% fraction of meiobenthic community densities and range between 407 and 2101 individuals 10 cm^−2^ (Gallucci et al. 2009; Oleszczuk et al. 2021). Due to their omnipresence, nematodes are one of the best‐studied meiofaunal clades in terms of biodiversity and ecology (Fonseca et al. 2010; Sharma et al. 2011; Grzelak et al. 2017), while less abundant groups such as copepods and kinorhynchs remain more obscure (Rosli et al. 2017; Chertoprud et al. 2018), and for some taxa, Arctic biodiversity data is lacking altogether. It is known, however, that abundances of nematodes and other meiobenthos tend to decrease with increasing depth (Grzelak et al. 2017; Oleszczuk et al. 2021), and even when boosting counts by including non‐metazoan eukaryotes, meiobenthos densities can be as low as 68–247 ind.·10 cm^−2^ in the depths of the central Arctic Ocean (Schewe and Soltwedel 1999). While nematodes and other meiobenthos typically comprise a small portion of standing benthos stocks in terms of biomass, high turnover rates lead to an outsized importance to benthic food webs (Gerlach 1971; Górska and Włodarska‐Kowalczuk 2017), and this ecological guild becomes relatively more important to benthic ecosystems with increasing water depth (Rex et al. 2006; Wei et al. 2010).

A multitude of environmental parameters associated with the benthic environment can affect the composition of deep‐sea meiobenthic communities, including water depth, food quantity and food quality (Soltwedel 2000), the latter of which is highly dependent on the seasonality of phytoplankton blooms. Much of the sublittoral meiobenthos is assumed to feed on a mix of microorganisms and sunken organic matter which includes sedimentary organic carbon as well as fresher phytodetritus (Majdi et al. 2020; Flo et al. 2025), and Arctic meiobenthic communities are likely food‐limited (Schnier et al. 2023; Silberberg et al. 2025). As such, many studies attest density and diversity increases in response to food pulses (Fonseca and Soltwedel 2006; Włodarska‐Kowalczuk et al. 2016; Soltwedel et al. 2018). For example, high primary production in the marginal ice zone along Eastern Greenland was found to enhance meiofauna abundances and the diversity of nematodes in a direct response to increased food availability (Fonseca and Soltwedel 2006). Similar enhancing effects have been documented in response to large food falls (Soltwedel et al. 2018). However, deep‐sea nematodes in polar regions have been observed to prefer feeding on bacteria over phytodetritus, suggesting a low dependency on freshly deposited bloom products (Ingels et al. 2010), and one colonisation experiment indicated that Arctic nematode densities do not increase in response to higher local levels of organic matter (Guilini et al. 2011). How the Barents Sea meiobenthic communities respond to seasonality is not well understood, but benthic macrofauna in the region do not show strong seasonal variability (Jordà‐Molina et al. 2023; Ziegler et al. 2023). Beyond levels and quality of food as structuring factors, abiotic aspects of the environment (such as grain size) are also known drivers of community compositions. For instance, soft‐bodied taxa such as flatworms tend to prefer coarse sediment such as sand, while, for example, kinorhynchs (‘mud dragons’) are characteristic of marine mud environments (Kristensen 2001; Ax 2008; Wernström, Smith, and Altenburger 2025). How the interplay between food and sediment composition contributes to the distribution and abundances of most taxa, however, remains poorly studied.

We describe sublittoral communities of metazoan meiobenthos along a transect of the northwestern Barents Sea and adjacent Nansen Basin and explore drivers of community structure variation through a synthesis of highly resolved environmental and taxonomic data. The Barents Sea is a marginal shelf sea to the Arctic Ocean where Arctic and Atlantic water masses meet to form a distinct and biologically productive oceanographic zone known as the Polar Front (Kolås et al. 2024). As a shelf sea, the Barents Sea system is characterised by tight benthopelagic coupling regarding the vertical sedimentation of phytoplankton bloom products to the sea floor (Wassmann et al. 2008; Wassmann and Reigstad 2011). Our primary objectives were (1) to uncover environment‐dependent patterns in the Barents Sea and Nansen Basin meiobenthic communities and (2) to provide a unique biodiversity dataset. We hypothesised that community structuring would be driven by spatiotemporal variation and that they would be influenced by food levels and quality as well as biotic and abiotic properties of the sediment. Due to their general abundance, we emphasised taxonomic identification of nematode, copepod and kinorhynch genera and species.

## 2 Methods

### 2.1 Geographical Context

**FIGURE 1:**
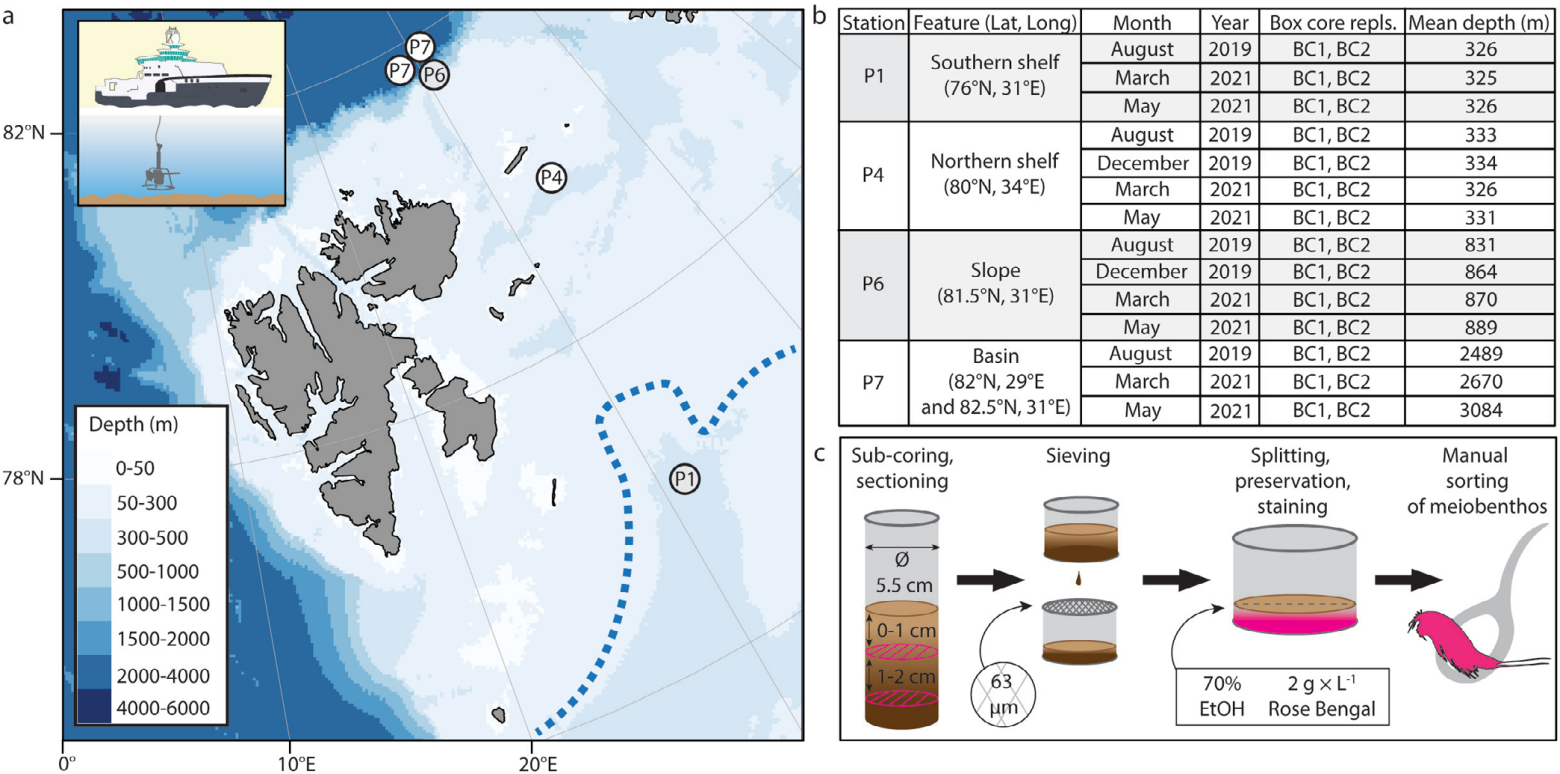
Study area and meiobenthological sampling approach. (a) Bathymetric map of the Barents Sea with benthic stations sampled with giant box corer (insert) by the Nansen Legacy project. Dotted line indicates approximate location of the Polar Front. (b) Overview of sampling events. (c) Schematic overview of the sample processing workflow.

Sediment samples were taken along a north–south transect with predefined stations in the Barents Sea onboard the R/V *Kronprins Haakon* according to protocols published by the Nansen Legacy project (The Nansen Legacy 2022). Ship‐based sampling was done in the months March, May, August and December during the years 2019–2021 (Gerland et al. 2022; Ludvigsen et al. 2022; Reigstad et al. 2022; Søreide et al. 2022). The stations covered a wide depth range of about 300–3000 m from the Barents Sea continental shelf (P1, P4) to the continental slope (P6) to the Nansen Basin (P7) of the Arctic Ocean (Figure 1a). The shelf stations were located south (P1) and north (P4) of the Polar Front. While care was taken to sample at the same coordinates each time, coordinates for some sampling events (particularly at station P7, see Figure 1a) varied slightly over seasons due to practical restraints related to sea ice cover and adverse weather. For the same reasons, no sampling was completed at stations P1 and P7 in the month of December. At each station, two box core replicates (bc1 and bc2, Figure 1b) of sediment were taken using a 50 × 50 cm giant box core. After removal of the overlying water in the box cores, one meiofauna subsample from each box core replicate was taken using a 5.5 cm diameter plastic sub‐core which was subsequently sectioned into two 1 cm thick fractions, covering the interval which tends to hold the most meiofauna (Rundell and Leander 2010). Sectioned sediment fractions (0–1 and 1–2 cm) were placed into separate 180 mL Joni containers, shaken gently to dissolve clumps, preserved with rose bengal‐stained 70% ethanol and stored at room temperature. After fixation, the samples were sieved with a 500 μm mesh to remove macrofauna and a 63 μm mesh on which meiobenthic animals were retained. Sieved samples were split in half, and one half was subjected to manual sorting and identification of metazoan meiobenthos (Figure 1c) while the other half was set aside for analysis of the foraminiferan community. Comprehensive environmental data recorded by the Nansen Legacy project members published previously (Jordà‐Molina et al. 2023; de Ricardo Freitas et al. 2024) were included in the analyses. As described in (Jordà‐Molina et al. 2023), sediment pigments were retrieved from sampled sediment using a sub‐core followed by acetone freeze‐extraction and centrifugation before measurements were taken with a fluorometer. Granulometry data for the 0–2 cm surface sediment layer (which was taken in August only) and total organic carbon content (TOC%) for the 0–1 cm surface sediment layer were used as previously published (de Ricardo Freitas et al. 2024; Sen et al. 2024).

### 2.2 Meiobenthos Processing Workflow

Meiobenthic metazoans from the sediment samples were manually sorted using an Irwin loop under a Nikon SMZ800 stereomicroscope. Animals were classified into higher phyletic groups, counted and preserved in 70% ethanol. Nematodes and kinorhynchs were identified to the lowest possible taxonomic levels at the Institute of Oceanology, Polish Academy of Sciences (IOPAN); copepods were identified at The Norwegian Institute for Water Research (NIVA); and cnidarian polyps were examined in detail at the University of Gothenburg. Less frequently encountered meiobenthic taxa were also counted and included in the dataset, but were not identified to lower taxonomic levels.

### 2.3 Data Analyses

All analyses were carried out using RStudio and the *vegan* package (R Core Team 2025). Principal component analysis (PCA) and ordination were used to visualise the relationship between the environmental variables (phaeopigments, mean chlorophyll a, TOC%, mean grain size, sand, clay and silt content, bottom salinity and water depth) reported in earlier works (Jordà‐Molina et al. 2023; de Ricardo Freitas et al. 2024). Prior to analyses of community structure, counts of meiobenthic taxa were converted into area abundances (ind. · 10 cm^−2^). To assess structuring effects on the meiobenthos community, we conducted a three‐way permutational multivariate analysis of variance (PERMANOVA). The analysis was based on a Bray–Curtis dissimilarity matrix calculated from log‐transformed abundance data of the following higher groups: nematodes, copepods, kinorhynchs, ostracods, annelids, cnidarians and nauplii while singletons and very infrequently identified taxa were omitted. We included three fixed factors: season (represented by the sampling months March, May, August and December), stations, and sediment fractions as well as their interaction, and added the two box core replicates as an additional term to account for variability between sampling occasions. Because weather conditions prevented sampling at stations P1 and P7 in the month of December, we imputed dependent variables for these sampling occasions using the impute_rlm function of the R package *simputation*. This function performs robust linear regression using M‐estimation to reduce the influence of extreme values when building the imputation model, and allowed us to apply the PERMANOVA model to the entire temporal gradient of higher taxon abundances (Table S1). Since the imputation was based on predictor variables (grain size, pigment levels, etc.) for which sediment‐fraction resolution was not available to us, the imputed values of the dependent variable (abundances) were the same for both sediment fractions. Factors were considered both as main effects and in interaction terms: all two‐ and three‐way interactions among the main factors were included. The significance of each term was tested separately using 9999 permutations. As environmental parameters such as grain size and TOC content were highly collinear with stations and seasons, they were not included in the PERMANOVA analysis. Instead, to permit attribution of environmental variable effects in detail, we also performed canonical correspondence analyses (CCA) of the environmental data and the relative abundances of nematodes, copepods and kinorhynch lower taxa respectively, and applied post hoc analysis of variance (ANOVA) to the output of each CCA analysis. These analyses were applied to a subset of the dataset's 20 most abundant nematode and copepod taxa hereafter referred to as T_20_, in order to focus on the main community contributors and preclude outsized influence caused by singletons.

## 3 Results

### 3.1 Sediment Properties in Brief

**FIGURE 2:**
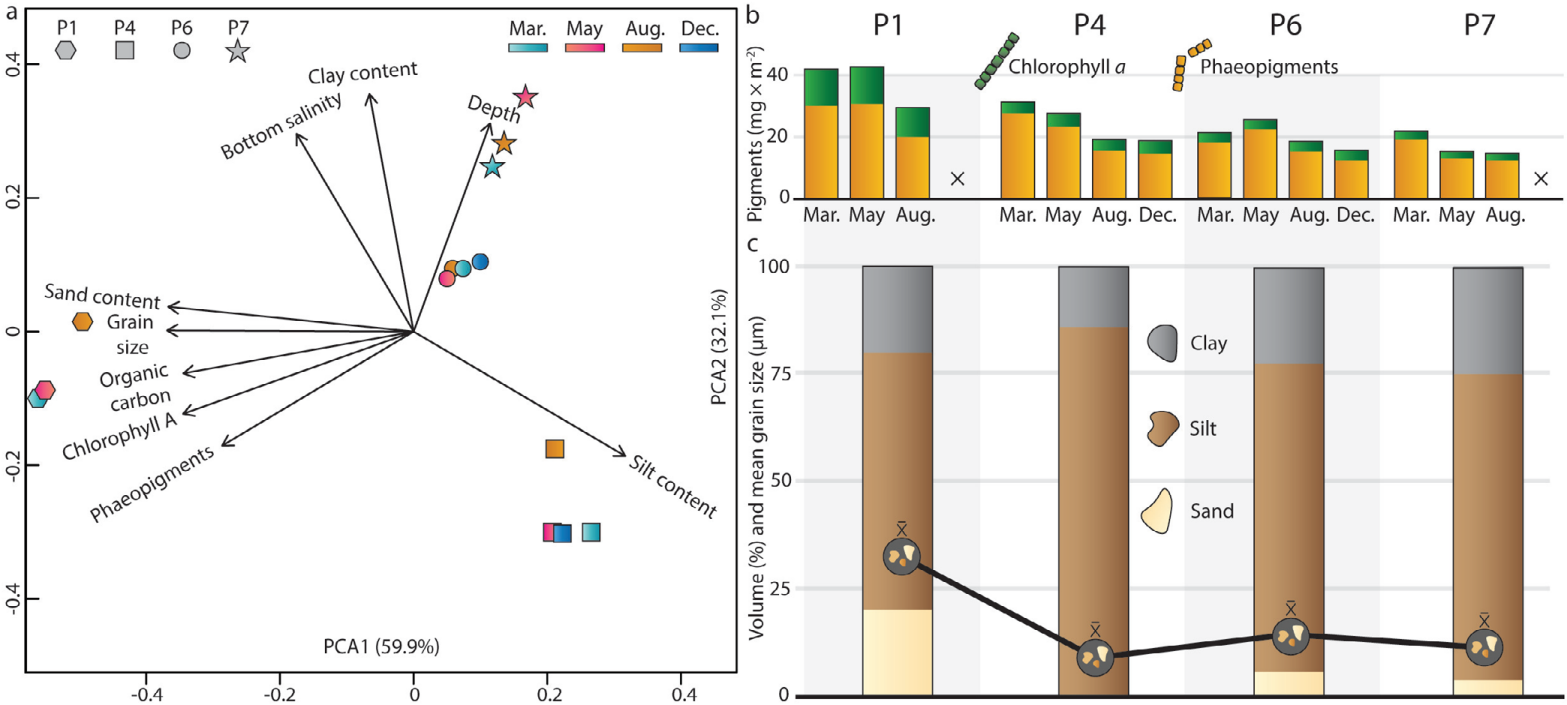
Overview of environmental variables relevant for meiobenthos. (a) Principal component analysis ordination of granulometric, sediment pigment, organic carbon and oceanographic data for the Barents Sea sampling stations. (b) Chlorophyll *a* and phaeopigment levels in the uppermost 2 cm of sediment for each station and season with × denoting missing data due to lack of sampling. (c) Granulometry for each station plotted on a common axis: each bar contains volume percentages of clay (top), silt (middle) and sand (bottom) for the uppermost 2 cm of sediment for each station, while the line indicates mean grain size in μm.

The physical environment for meiobenthos along our Barents Sea transect is characterised by substantial heterogeneity between the sampling locations (Figure 2a). Chlorophyll *a* and phaeopigment levels varied more between stations than between seasons (Figure 2b). Organic carbon values ranged between 1.3% and 2.1%, with the highest level recorded on the shelf compared to the lower levels observed at the slope and in the Nansen Basin. Mean sediment grain sizes ranged between 9.1 and 32.5 μm, and the proportions of silt, sand and clay differed between stations, although silt was the predominant sediment type at all stations (Figure 2c).

### 3.2 Community Structure of the Barents Sea Meiobenthos

**FIGURE 3:**
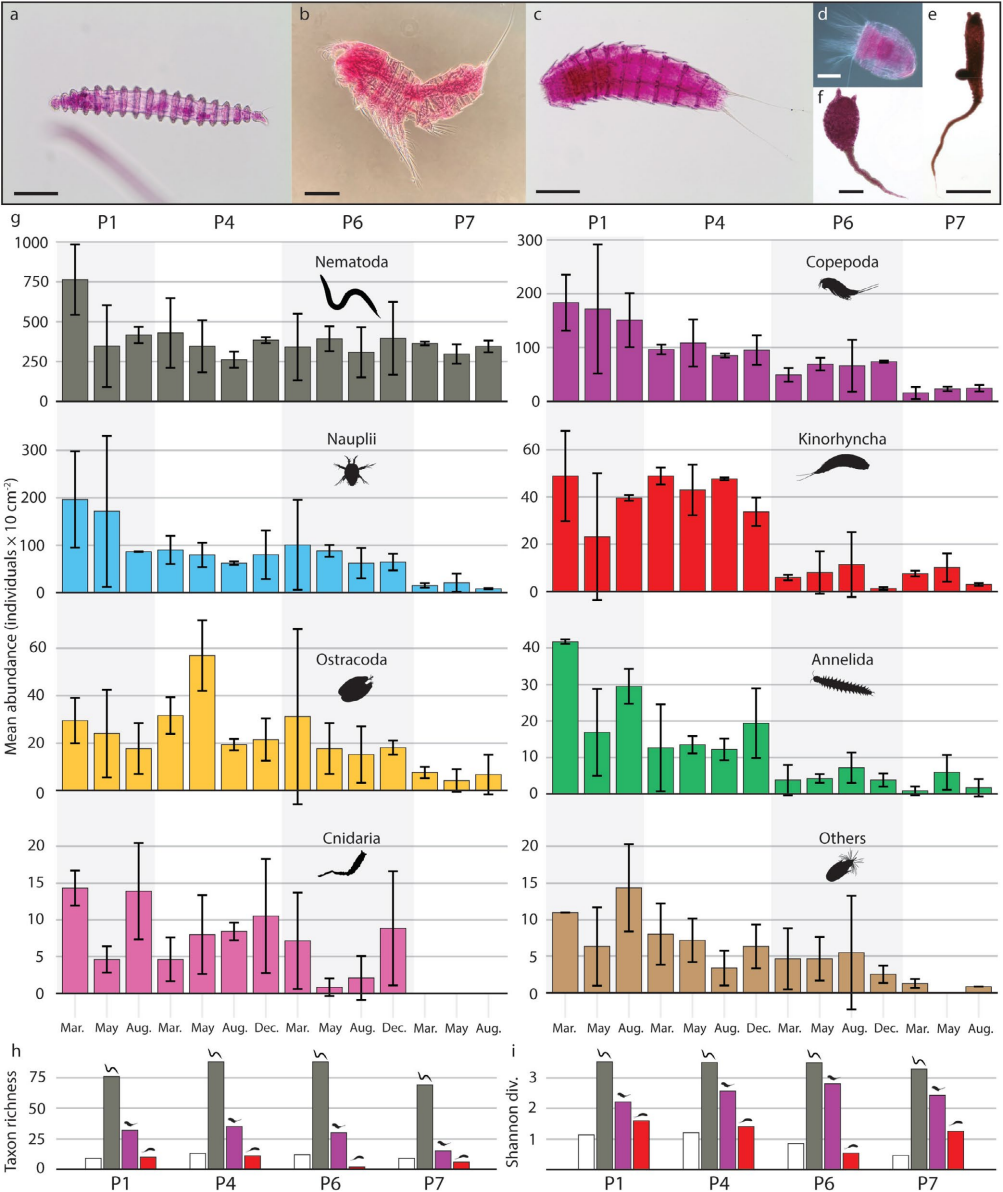
Abundances and diversity of meiobenthic metazoans in the Barents Sea and Nansen Basin. (a) The nematode *Desmoscolex* sp., scale bar = 50 μm. (b) The harpacticoid copepod *Heteropsyllus* sp., scale bar = 100 μm. (c) The cyclorhagid kinorhynch *Echinoderes arlis* , scale bar = 100 μm. (d) The loriciferan *Rugiloricus* sp., scale bar = 50 μm. (e, f) *Plotocnide borealis* polyps with typical (e) and deviant (f) morphology. (g) Arithmetic mean abundances of higher groups with standard deviations in the 0–2 cm of sediment for the two replicates. (h, i) Taxon richness (h) and Shannon diversity index (i) values for higher taxa (white bars), nematode genera (grey bars), copepod species (purple bars) and kinorhynch species (red bars) from the bc1 replicate of all seasons and stations combined.

Across the transect, we identified specimens of 12 higher‐level taxa and additional indeterminate organisms, nauplii and other larvae, listed roughly in order of falling abundance: nematodes, copepods, kinorhynchs, ostracods, annelids, bivalves, cnidarians, tardigrades, acarids, peracarids (including isopods, tanaids and cumaceans), echinoderms and loriciferans (Figure 3a–f). We observed a clear trend of higher abundances (Figure 3g) at the shelf stations (P1, P4) than at the slope (P6) and in the Nansen Basin (P7), and in total, we recorded 9 higher‐level taxa on the southern shelf, 12 on the northern shelf, 12 on the slope, and 8 in the basin. Whole‐community mean abundances for the combined sediment 0–2 cm fractions ranged from as high as 1574 ind. · 10 cm^−2^ at station P1 in March to below 284 ind. · 10 cm^−2^ at station P6 in March. Taxon richness and Shannon diversity of higher‐level taxa were generally higher at the shelf and slope than in the Nansen Basin (Figure 3h,i). Nematode taxon richness was highest on the northern shelf and slope, while Shannon diversity was similar across the transect with a slightly lower value in the Nansen Basin. Copepod taxon richness decreased in the Nansen Basin as compared to the shelf and slope, while Shannon diversity rose somewhat at the slope and basin stations compared especially to the southern shelf. Kinorhynch taxon richness also decreased with depth, while Shannon diversity was generally higher on the shelf than in the basin, and lowest at the slope.

**FIGURE 4:**
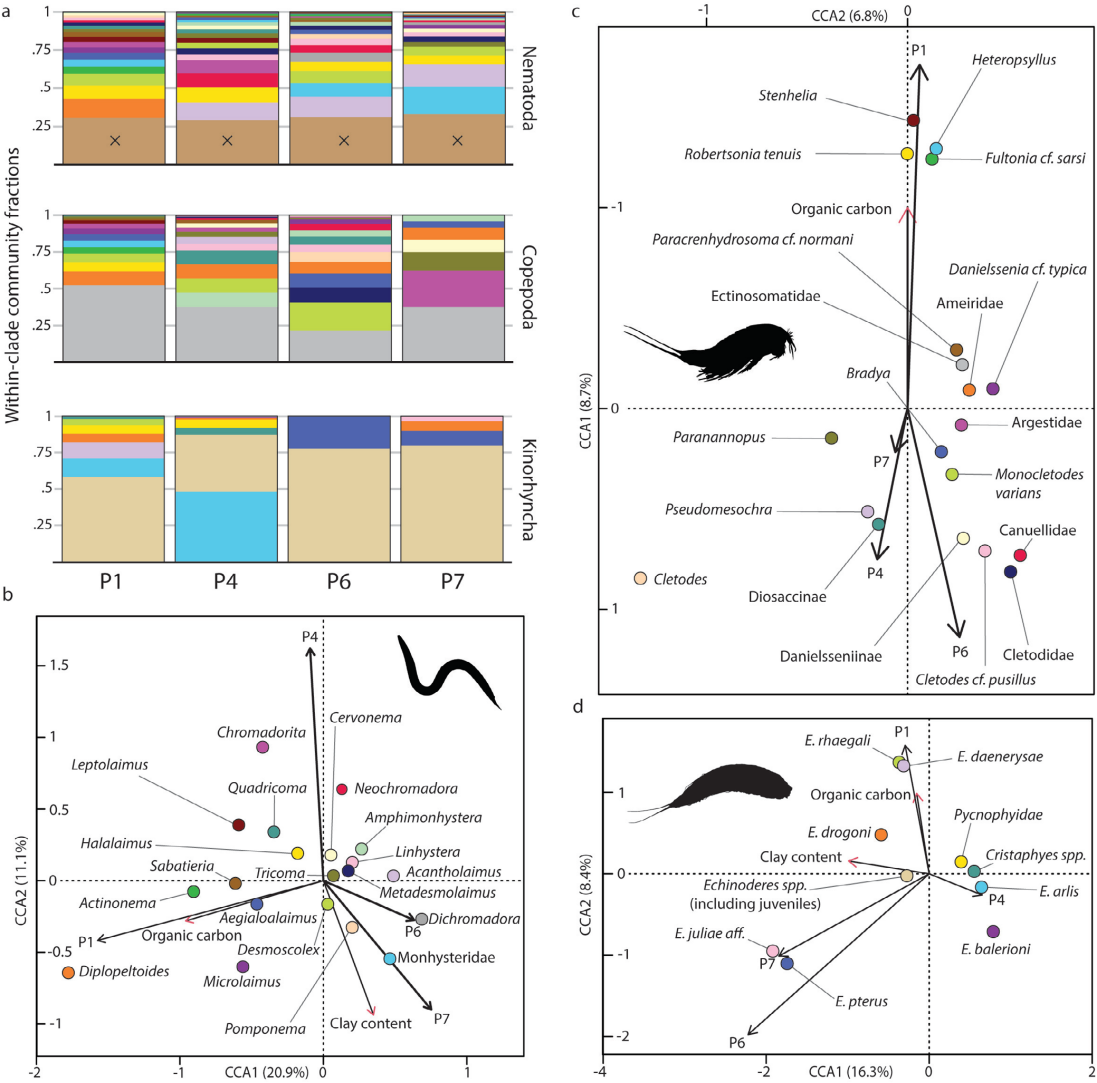
Within‐clade community fractions and canonical correspondence analysis ordinations for relative abundances of nematodes, copepods and kinorhynchs in relation to environmental parameters with significant influence. (a) Community fractions comprised by the identified kinorhynch taxa and the nematode and copepod T_20_ subsets, shown in order of highest frequency. Bars marked × denote lower‐abundance taxa. (b) CCA of nematode community T_20_ subset (*R* ^2^ = 0.32) in relation to organic carbon (*p* = 0.001) and clay content (*p* = 0.001). (c) CCA of copepod community T_20_ subset (*R* ^2^ = 0.07) in relation to organic carbon (*p* = 0.05). The outlier *Pseudobradya* is obscured from the plot. (d) CCA of kinorhynch community (*R* ^2^ = 0.25) in relation to organic carbon concentration (*p* = 0.039) and clay content (*p* = 0.001). *Cristaphyes glaurung* and *Cristaphyes dordaidelosensis* were ordinated collectively as *Cristaphyes* sp.

Nematodes were the numerically dominant group of benthic metazoan meiofauna recovered (47%–90% of the communities) with mean abundances for the two replicates ranging between 258 and 747 ind. · 10 cm^−2^ for the 0–2 cm of sediment (Figure 3g). Mean nematode abundances were lowest at the northern shelf (P4) in August and highest on the southern shelf (P1) in March. We identified 143 genera in addition to specimens which could only be determined to family level, with 78 taxa recorded on the southern shelf, 90 on the northern shelf and slope respectively, and 70 in the basin. Particularly abundant genera of the assemblage included *Diplopeltoides*, *Halalaimus*, *Desmoscolex*, *Acantholaimus* and various representatives of the family Monhysteridae (Figure 4a). CCA analysis of the nematode community composition and environmental variables uncovered major axes of variation associated with organic carbon levels and clay content (Figure 4b). ANOVA analysis of the CCA output identified organic carbon levels (*p* = 0.001) and clay content (*p* = 0.001) as variables which significantly contributed to variation in the nematode community dataset.

Copepods were the second most numerous taxonomic group (3%–24% of the communities) with mean abundances for the two replicates ranging from 13 to 180 ind. · 10 cm^−2^ for the 0–2 cm of sediment. Mean copepod abundances were lowest in the Nansen Basin (P7) in March and highest on the southern shelf (P1) in March. We identified 37 genera, some of which contained several discernible species, with 33 taxa recorded on the southern shelf, 36 on the northern shelf, 31 at the slope and 16 in the basin. Harpacticoid copepods were the most abundant group recovered, followed by Canuelloida and Cyclopoida. Shannon diversity of copepods was highest on the slope at P6 and lowest at the southern shelf (P1), while taxon richness was highest on the northern shelf (P4) and lowest in the basin (P7) (Figure 3h,i). While indeterminate members of the Ectinosomatidae were one of the most abundant copepod lower taxa at all stations and seasons, genera such as *Heteropsyllus* and *Monocletodes* were also frequently encountered (Figure 4a). Ectinosomatidae were dominant except at P6 and P7, where Cletodidae or Pseudotachididae (Danielsseniinae) were slightly more numerous. The southern shelf was distinguished by genera like *Heteropsyllus*, *Robertsonia* and *Fultonia*, which were absent further north. The cletodid *Monocletodes varians* was common at all stations except P7. Miracidae (Diosaccinae and Stenheliinae) were common at P1 and P4, rare at P6 and absent at P7. Argestidae occurred at all stations, with relative dominance highest in the basin (P7) but also common on the shelf at P1. Cletodidae were common on the shelf stations and dominant at the slope (P6) but not recorded at P7. CCA analysis of the copepod community and environmental variable datasets and subsequent ANOVA analysis indicates that the organic carbon level (*p* = 0.05) significantly contributes to variation in the copepod community dataset, with most variation occurring along CCA axis 1 (Figure 4c). One specimen of the highly specialised, ectoparasitic multicrustacean clade Tantulocarida was identified parasitizing on a *Bradya* sp. copepodite.

Kinorhynchs comprised up to 10% of the communities with mean abundances for the two replicates reaching up to 49 ind. · 10 cm^−2^ for the 0–2 cm of sediment. Mean kinorhynch abundances were lowest at the slope (P6) in December and highest on the northern shelf (P4) in March. Specimens were recovered at all stations, and taxa were distributed unevenly across the two main clades Allomalorhagida and Cyclorhagida, with most specimens belonging to the latter. We recovered 15 taxa in total, with 11 taxa at the southern shelf, 12 on the northern shelf, 2 at the slope and 7 in the basin. Cyclorhagid kinorhynchs in the genus *Echinoderes* were represented by 7 identified species and this was theoverall most abundant kinorhynch genus recorded by us (Figure 4a). A few cyclorhagids, like *Echinoderes pterus*, seem characteristic for deep waters and were present only at stations P6 and P7. Two allomalorhagid species, *Cristaphyes glaurung* and *Cristaphyes dordaidelosensis*, were identified in addition to unidentified specimens of Pycnophyidae. CCA analysis and ordination uncovered variation in the kinorhynch dataset along an axis defined by organic carbon levels, aligned with axis 2, and clay content, aligned with axis 1 (Figure 4d). ANOVA analysis of the CCA output identified clay content (*p* = 0.001) and organic carbon levels (*p* = 0.039) as variables which significantly contributed to variation in the kinorhynch dataset.

Ostracods comprised up to 9% of the communities and a maximum of 56 ind. · 10 cm^−2^ in mean abundances for the two replicates for the 0–2 cm of sediment. Mean ostracod abundances were lowest at the basin station (P7) in May and August, and highest on the northern shelf (P4) in May. Annelids constituted up to 4% of the communities and as many as 42 ind. · 10 cm^−2^ for the 0–2 cm of sediment. Annelids were absent from the basin (P7) samples in March and August, and had the highest mean abundances on the southern shelf (P1) in March. Nauplii made up 2%–21% of the communities and ranged between 8 and 183 ind. · 10 cm^−2^ in mean abundances for the two replicates for the 0–2 cm of sediment. Mean naupliar abundances were lowest at the basin station (P7) in August, and highest on the southern shelf (P1) in March.

Meiobenthic cnidarians in the form of aplanulate hydrozoan polyps constituted up to 2% of the communities and were primarily represented by *Plotocnide borealis* (Figure 2e–f), which comprised 71 out of 81 examined specimens and ranged between 4 and 14 ind. · 10 cm^−2^ in mean abundances for the 0–2 cm of sediment of the two replicates. We observed notable variation in body length (0.47–2.49 mm) and morphology of the caulus and tentacles in the *Plotocnide* specimens, but only one reproductive specimen was recovered, and no specimens were found at the basin station (P7). The remaining hydrozoan specimens were identified as indeterminate aplanulates, in addition to a putative planula larva.

Other taxa such as bivalves, tardigrades, acarids, peracarids and echinoderms collectively comprised up to 2% of communities. Two loriciferan specimens identified as *Rugiloricus* sp. were obtained from the 0–1 and 1–2 cm sediment fractions of station P6 (Figure 2d). The whole taxonomic dataset generated in this study is available at the Global Biodiversity Information Facility (Wernström, Bluhm, et al. 2025).

### 3.3 Spatiotemporal Structuring Effects on Meiobenthic Communities

Our three‐way PERMANOVA analysis indicated a highly significant structuring effect of stations and sediment fractions on meiobenthic communities on the level of higher groups, explaining 36% and 18% of the variation respectively (Table 1). We also found support for a seasonal effect with the sampling month accounting for an additional 7% of dataset variability, as well as for the significant interaction of season and station terms which accounted for another 1%.

**TABLE 1:** PERMANOVA summary statistics.

Terms	Degrees of freedom	Sum of squares	*R* ^2^	Pseudo‐*F*	*p*
Season	3	0.12	0.07	3.9	**0.0017**
Station	3	0.64	0.36	21.9	**0.0001**
Sediment fraction	1	0.32	0.18	32.1	**0.0001**
Box core replicate	1	0.02	0.01	2.5	0.0785
Season × Station	9	0.18	0.01	2	**0.0211**
Season × Sediment fraction	3	0.05	0.03	1.8	0.0954
Station × Sediment fraction	3	0.04	0.02	1.2	0.3064
Season × Station × Sediment fraction	9	0.1	0.06	1.2	0.2997

*Note:* Meiobenthos community structure on the level of log‐transformed abundances of higher taxa tested for differences between the three fixed factors of stations, seasons and sediment fractions as well as their interaction, and the two box core replicates (bc1 and bc2) as an additional term with regression imputation of December data and 9999 permutations. Statistically significant terms shown in bold font.

## 4 Discussion

### 4.1 Patterns of Abundances and Biodiversity in Barents Sea Meiobenthic Metazoans

Our observation of a depth‐dependent decrease in community‐wide mean abundances confirms the findings of previous studies in the region (Pfannkuche and Thiel 1987; Oleszczuk et al. 2021; Schnier et al. 2023). The apparent depth gradient continues into the central Arctic ocean, where (Schewe and Soltwedel 1999) reported community‐wide abundances as low as 68–247 ind. · 10 cm^−2^ (unlike our data, this also included unicellular eukaryotes). Higher abundances on the shelf (P1 and P4) than at the slope (P6) and basin (P7) stations may be related to the larger quantities and higher quality of food available to these communities, as shown by, for example, Oleszczuk et al. (2021). In terms of biodiversity, we observed a community‐wide trend of higher taxon richness at the northern shelf and at the continental slope, which corroborates the realisation that slope habitats can be surprisingly rich in benthic biodiversity (Gage 2002; Danovaro et al. 2009; Kaiser et al. 2011; Levin and Sibuet 2012) and shows that this can also hold true for meiobenthic taxa. Biodiversity as measured by the Shannon index of higher groups generally followed the transect depth gradient, with higher values on the continental shelf, a lower value at the slope and the lowest value in the basin. This observation reflects a depth‐dependent impoverishment of whole‐community biodiversity on the level of higher taxa (Figure 3i, white bars), and confirms the well‐documented phenomenon of general decreases in diversity of marine taxa with depth (Costello and Chaudhary 2017). However, the picture becomes more complex when examining nematode, copepod and kinorhynch diversity in detail.

As for nematodes, the decrease in taxon richness towards the basin station is consistent with the decrease in overall diversity with increasing depth documented, for example, in the nearby Laptev Sea (Vanaverbeke et al. 1997). At all stations, the T_20_ subset of taxa made up the majority of their communities by abundance at each station, while many low‐frequency taxa accounted for a substantial proportion of total taxon richness. This indicates that local environmental conditions may favour numerical dominance of some taxa, but possibly also reflects life‐history traits. The assemblage of nematodes encountered contained several genera which have also been documented in nearby areas such as Kongsfjorden (Svalbard) and the Fram Strait (Urban‐Malinga et al. 2005; Grzelak et al. 2017). All of the most dominant genera recently recorded at the HAUSGARTEN observatory in the Fram Strait (Schnier et al. 2023) were also recorded by us, and most occupied similarly dominant positions by abundance in the dataset. An indication that the biodiversity of the nematode communities is dependent on the spatial aspect comes from the fact that taxonomic composition varied between stations. In terms of individual taxa, we saw that, for example, the genus *Acantholaimus* was highly prevalent at the northern shelf and slope stations, supporting asimilar pattern of increasing abundances of *Acantholaimus* with depth repeatedly reported from the Fram Strait (Grzelak et al. 2017; Schnier et al. 2023). *Microlaimus*, a genus which has been encountered in deep sea sediments, for example, at HAUSGARTEN (Grzelak et al. 2017), was recovered by us not only in the Nansen Basin but also as a substantial contributor to the community abundance on the southern shelf. This wide distribution may indicate that the specimens from each site belong to different species, which is unsurprising for a genus as diverse and widely distributed as *Microlaimus* (Esteves et al. 2025). Although high relative abundances of genera such as *Thalassomonhystera* and *Daptonema* have been reported as characteristic of slope environments elsewhere (Vanreusel et al. 2010; Grzelak et al. 2017), we did not see a particularly strong contribution from them at the slope station P6.

Copepod communities differed substantially in composition between stations. Harpacticoids made up the absolute majority of copepod diversity, which reflects their well‐known dominant position in meiobenthic communities (Dahms and Qian 2004), where they are often the most numerous arthropods. In a similar fashion to the nematodes, the T_20_ subset of copepod taxa made up a large portion of the within‐clade abundance, while a large proportion of the other encountered taxa are rare at our sampling stations, but may be more abundant elsewhere. Interestingly, copepod taxon richness showed a clear decrease with depth (Figure 3h), while their Shannon diversity was fairly stable but highest on the slope station and lowest at the southern shelf, indicating that while there are fewer taxa at depth, the evenness of copepod assemblages is rather constant or increasing with depth. Like the general pattern, the 0–1 cm sediment layer contained most individuals of nearly all copepod taxa. An exception was Paranannopus spp., where 15 out of 17 individuals occurred in the 1–2 cm fraction. Most of the 37 taxa identified in our samples are also known from neighbouring areas such as the Kara Sea (Garlitska et al. 2019) and the Laptev Sea (Chertoprud et al. 2018), and appear to be widely distributed in the Arctic. The material holds potential for more detailed information than presented in this study due to limitations of time and resources. Copepod species richness is certainly underestimated, and several taxa clearly harboured unidentified and possibly new species, for example, Danielsseninae, Cletodidae and Argestidae.

Kinorhynch communities at all stations were dominated by species of the genus *Echinoderes*, which is widely distributed with at least 20 species recorded in the Arctic (Grzelak and Sørensen 2018, 2019b), out of which we have documented 7 in addition to some indeterminate specimens. It is interesting to note that kinorhynch taxon richness was highest on the northern shelf, reflecting the general trend shown by several other taxa studied herein, but that it was lowest at the continental slope. Shannon diversity values were fairly similar when comparing the shelf and the basin but were substantially lower at the slope station P6, indicating dominance by a few taxa. In fact, we only documented *Echinoderes pterus* and indeterminate members of the same genus at the slope station. A bathymetrically wide distribution pattern previously documented for *E. pterus* (Yamasaki et al. 2018), with specimens recorded between 675 and 4403 m, is corroborated by our identification of this taxon exclusively at the slope and basin stations. Generally, kinorhynch communities along our transect resemble those previously documented from Svalbard and the Arctic Ocean north of Svalbard (Grzelak and Sørensen 2018, 2019b), with *Echinoderes arlis* being particularly abundant in shelf communities. We also recorded *E. drogoni* both on the shelf and in the deep sea, consistent with earlier findings (Grzelak and Sørensen 2019b). However, not all taxa previously reported from Svalbard fjords were encountered in our study, for example, *Echinoderes aquilonius* or *Echinoderes eximus* were absent, supporting the idea that Arctic kinorhynch communities vary depending on environmental conditions and their structure may differ considerably between fjords and open‐water systems (Grzelak and Sørensen 2019a). Allomalorhagid kinorhynchs were less abundant than cyclorhagids, and we documented two out of the three *Cristaphyes* species described from Svalbard waters (Grzelak and Sørensen 2019b), *C. glaurung* and *C*. *dordaidelosensis*. However, we did not identify specimens of *C. scatha* nor the other known Arctic genera of allomalorhagids, *Krakenella* and *Pycnophyes* (Sørensen and Grzelak 2018). However, we did recover indeterminate and juvenile pycnophyid specimens.

Among the less frequent meiobenthic taxa, our identification of the loriciferan *Rugiloricus* sp. seems to represent a first record of this somewhat elusive phylum in the Barents Sea, with the closest records to our knowledge being from the area around Jan Mayen and from the Laptev Sea (Gad and Arbizu 2005). Species‐level identification was precluded by the reliance of our sampling approach on direct ethanol preservation, which degrades taxonomic characters in Loricifera and many other groups. We deem it likely that other loriciferan taxa remain undocumented in the region. Additionally, we have provided abundance data for the hydrozoan *Plotocnide borealis* with unprecedented seasonal and spatial resolution. No *Plotocnide* polyps were recovered in the deep‐sea Nansen basin station P7, indicating that they are associated with shallower environments such as the continental shelf. Candidates for taxonomic placements of the polyps we could not identify to species or genus level could be some of the known species which lack described polyps, for example, *Margelopsis hartlaubii* and *Euphysa tentaculata* .

### 4.2 Spatiotemporal Variation and Sediment Properties Contribute to Community Structuring

In line with previous treatments of meiofauna responses to food availability (Soltwedel 2000) we expected that the seasonal nature of primary production in the High Arctic could drive cyclical changes in Barents Sea meiobenthos communities to some extent. This expectation is supported by a wealth of studies which indicate that the quantity and quality of food impact abundances and biodiversity of the meiobenthos at least within restricted spatiotemporal intervals (Pfannkuche and Thiel 1987; Soltwedel 2000; Fonseca and Soltwedel 2006; Hoste et al. 2007; Ingels et al. 2010; Gambi et al. 2013; Górska et al. 2014). Our PERMANOVA analysis confirms this notion, but the amount of variation explained by the model is substantially lower than that explained by the spatial differences between stations and sediment slices. The apparent limited influence of seasonally driven variation in Barents Sea meiobenthos community structures is, as such, congruent with the lack of strong seasonality signals in macrobenthos abundance and trophic structure in the same region (Jordà‐Molina et al. 2023; Ziegler et al. 2023). This solidifies the notion that seasonal variability of High Arctic primary producer blooms generally does not translate to strong structural cyclicity in the benthic community, even if some seasonal variability is present and attestable. Since the interaction of station and season terms was a significant contributor to dataset variability, it implies that the seasonal patterns in community composition are not consistent across stations. Reasonably, they are less pronounced in the deep sea than in shallower areas since more of the sedimenting organic matter can be degraded during the long process of sinking to the sea floor at great depth (Soltwedel 2000). However, it is important to note that our approach of sampling during the four different months of March, May, August and December has its limitations in capturing seasonal variability, for example, due to interannual differences in the timing of phytoplankton blooms. Thus, the seasonal signal could be more prominent in a dataset resolved to, for instance, each month of the year. However, we assess that obtaining such resolution for a meiobenthos dataset in our remote region of study is currently unfeasible. Our data show that nematode, copepod and kinorhynch community structures are all significantly influenced by levels of organic carbon in the sediment. However, we could not find support for a relationship of any taxon to sediment pigment levels, and it is possible that more degraded organic matter is the primary food source for much of the Barents Sea meiobenthos. In line with this, some deep‐sea polar nematodes are known to be ‘picky eaters’ and feed preferentially on bacteria and degraded organic matter rather than phytodetritus (Ingels et al. 2010), and we consequently speculate that stable organic carbon levels may contribute to community stability. Organic carbon levels in the northern Barents Sea show no signs of seasonal variability (de Ricardo Freitas et al. 2024), and may act as a food bank for meiobenthic taxa. We note that seasonal effects on macrofauna in the Barents Sea are demonstrably also weak (Jordà‐Molina et al. 2023) which indicates that benthic communities in this region may be partially buffered from seasonality in ocean surface processes. Supporting the notion of a stabilising effect of TOC levels on meiobenthic communities, earlier studies have also documented sedimentary carbon levels as an important factor, for example, Arctic deep‐sea nematodes at HAUSGARTEN in the Fram Strait (Grzelak et al. 2017). However, it should be noted that most of the previous studies conducted in the Arctic provide information for only one season, or describe very specific situations such as in situ experiments (Guilini et al. 2011) and their results may not be directly comparable to ours.

Spatial variation exerted a consistent influence on the meiobenthos community structure both on the level of higher groups and on the level of lower taxa. It fell into three spatial scales: the very small scale (cm) of sediment depth, the intermediate scale of box core replicates (m), and the regional scale (km) between stations. On the smallest scale, we found statistically significant differences between higher group community structures in the upper (0–1 cm) and lower (1–2 cm) sediment fractions, with the upper fractions holding more fauna by abundance. Non‐nematode taxa were generally more abundant in the uppermost sediment fraction, which mirrors the results of earlier studies on communities across sediment depths (Gallucci et al. 2009; Ingels and Vanreusel 2013). Nematodes tended to increase in relative dominance in the lower sediment fraction, which might be connected with their slender body shapes and higher mobility within the sediment. The strong influence of sediment depth on community structure in our study is supported by previous research that accounts for vertical zonation (Górska et al. 2014; Rosli et al. 2017) and aligns with the idea that the upper sediment layers typically support a denser and more diverse fauna than deeper layers. On the intermediate scale, we had expected that variation between box cores taken a few meters apart could yield different community structures based on studies which attest such variation at much smaller distances (Gallucci et al. 2009; Ingels et al. 2011; Schratzberger and Larcombe 2014). As the two box core replicates did not produce significantly differing community structures (Table 1), we surmise that the stations sampled by us represented consistent environments, and lack the horizontal, centrimetric community variability attested elsewhere. On a regional scale, we observed a clear gradient in the meiobenthos dataset ultimately related to the different water depths and habitat presented by the sampling stations, which consistently yielded significant differences in community structure. As a rule, abundances were higher on the continental shelf and lower in slope and basin. Grain size is known to be a major structuring variable for meiobenthos, including nematodes (Smol et al. 2020), and the differing sediment granulometry between stations partially drives the regional variation observed, for example, for nematode and kinorhynch communities. Meanwhile, it is possible that copepods tend to be less affected by grain size because they generally have epibenthic rather than interstitial ecologies.

### 4.3 Limitations

Most of the limitations in this study stem from the challenges of marine biological fieldwork in the Arctic or are the result of samples and gear being shared on the highly interdisciplinary Nansen Legacy cruises. Box corers are not an ideal choice for sampling deep‐sea meiofauna, and unlike more suitable multicorers, may create bow waves upon impact and potentially wash away meiobenthos in the most superficial sediment fraction (Shirayama and Fukushima 1995). We opted for a comparatively narrow diameter of the sub‐core (5.5 as opposed to 10 cm which is the standard in metazoan meiobenthos deep‐sea research) and sieved sediment over a 63 μm rather than 32 μm mesh. This approach may have led to a loss of a proportion of the smallest animals, and comparisons of our abundances to those of other studies need to account for this. For taxonomic identification purposes, other approaches than direct ethanol preservation of samples could have been beneficial, and while it would be optimal to have ‘fresh’ environmental data associated with each sampling event, it was not practically and economically feasible. It is also important to note that other environmental parameters not considered herein, such as redox potential or O_2_ levels, could affect communities. Year‐to‐year variability could also have had an influence, considering that August and December samples were collected in 2019, while March and May samples were, due to the COVID‐19 pandemic, collected over a year later. In terms of statistical analysis, our dataset lacked December values for two of the four stations due to weather and sea ice conditions. To allow consideration of a full temporal gradient, we applied regression imputation of the missing December values for the two stations affected. Naturally, this approach has its drawbacks, including the imputation being based on environmental data without sediment fraction‐level resolution, and we have therefore approached the topic of seasonality in our dataset with some caution. As a closing remark, we would like to note that while our interdisciplinary sampling approach may have resulted in overall lower meiofauna abundances as compared to other studies, it was consistent throughout the project.

### 4.4 Future Recommendations and Outlook

We have explored environmental and spatiotemporal patterns of metazoan meiobenthos community structures in the Barents Sea, and provided novel insights into the biodiversity of deep‐sea meiofauna. Our work represents one of the most comprehensive biodiversity surveys of Arctic meiobenthos thus far, and points towards a strong relationship between meiobenthic community structures and environmental variables of the heterogeneous sea floor. As climate change threatens polar benthic ecosystems with profound and irrevocable alterations, the biodiversity knowledge generated herein may contribute to a benchmark of community compositions for the UN Ocean decade. Additionally, the knowledge of spatiotemporal and environmental structuring effects on meiobenthic metazoans in the Barents Sea may prove informative for forecasting how benthic ecosystems will be impacted by alterations to, for example, sedimentary organic carbon driven by climatic changes in the seasonal primary production. Future research in the area would benefit from establishing longer time‐series and from expanding the scope of taxonomic groups considered.

## Author Contributions

**Joel Vikberg Wernström:** data curation (lead), formal analysis (lead), investigation (lead), software (lead), visualization (lead), writing – original draft (lead), writing – review and editing (lead). **Bodil A. Bluhm:** conceptualization (lead), methodology (lead), project administration (lead), writing – review and editing (equal). **Katarzyna Grzelak:** data curation (equal), investigation (equal), validation (equal), writing – review and editing (equal). **Anders Hobæk:** data curation (equal), investigation (equal), writing – review and editing (equal). **Doris Björling:** data curation (equal), investigation (equal), writing – review and editing (equal). **Andreas Altenburger:** conceptualization (equal), project administration (supporting), resources (lead), supervision (lead), writing – review and editing (equal).

## Conflicts of Interest

The authors declare no conflicts of interest.

## Supporting information


**Table S1:** Abundances of higher meiobenthic taxa in the Barents Sea.

## Data Availability

Abundance data has been provided as Table S1 to this article. Taxonomic data has been made publicly available through the Global Biodiversity Information Facility (https://doi.org/10.15468/vj8wxt).
